# Reply to Luger, “Why Is It So Hard to Find Persistent Borreliella burgdorferi?”

**DOI:** 10.1128/mbio.02169-22

**Published:** 2022-08-22

**Authors:** Felipe C. Cabello, Monica E. Embers, Stuart A. Newman, Henry P. Godfrey

**Affiliations:** a Department of Pathology, Microbiology and Immunology, New York Medical College, Valhalla, New York, USA; b Division of Immunology, Tulane National Primate Research Center, Tulane University Health Sciences, Covington, Louisiana, USA; c Department of Cell Biology and Anatomy, New York Medical College, Valhalla, New York, USA; University of Texas Health Science Center at Houston

**Keywords:** *Borreliella burgdorferi*, Lyme disease, antimicrobial tolerance, bacterial persistence, persistence, post-Lyme disease syndromes, posttreatment syndromes

## REPLY

We thank Dr. Steven W. Luger for his interest in our review and his clinically related comments. We have responded to them below.

Dr. Luger is correct in noting that the number of cases mentioned in our review is based on insurance data (1). We agree that the true number of cases is likely to lie somewhere between 30,000 and 476,000 (2). It could certainly be greater, since cases of the disease are increasing in number and geographical distribution. This increase is important to any discussion of posttreatment Lyme disease syndrome (PTLDS), since at least 10 to 20% of cases of Lyme disease are currently thought likely to develop PTLDS (1, 3).

Lyme disease has existed in Europe for over 100 years, and as we discussed (1), many of the first clinical reports showing persistence of borrelia after adequate treatment were from Europe (4). One possible reason for the lack of past recognition of PTLDS is that its chronic symptomatology was ascribed to other etiologies. This is clearly the case for acrodermatitis chronica atrophicans and erythema migrans before Borreliella afzelii was identified as their main cause (5).

It is not really surprising that most tick bites in an area with a very high rate of tick infection with B. burgdorferi do not result in transmission of infection, since studies have shown that the transmission rate from tick to human is only 1 to 2% (6). As transmission from infected ticks to human hosts depends on multiple factors, the 36 to 48 h of tick attachment needed for efficient spirochete transmission must also play a role (6, 7).

We described multiple experiments in two preferred models of Lyme disease, mice and macaques (1). In both models, borrelial persistence occurred after antimicrobial treatment, often in the dense collagenous matrices of tissues. In these potentially immunologically privileged sites, borrelia could evade the humoral immune response and be simultaneously pathogenic and undiagnosable (8–11). Because comparable studies in human patients can only be performed on autopsy tissues or surgical discards, it is currently unknown if persistent borrelia are located in such sites in patients with PTLDS or if these borrelia can stimulate or evade antiborrelial immune responses.

The statement that Western blots of patients with PTLDS do not generally show “expansion in the number of bands” wrongly assumes that immunological responses to borrelia of patients with PTLDS are restricted to antiborrelial antibodies detectable by this assay. Because patients who go on to develop PTLDS generate fewer antibody-producing plasmablasts than patients who clinically recover after treatment (12), such differences might not be detectable on standard Western blots. In addition, there are also many other types of antiborrelial immune responses (including regulatory ones) not detectable on Western blots yet documented to occur in these two patient groups (13).

Despite Dr. Luger’s assertion, evidence for “persisting spirochetes” in patients is not rare. As we discussed (1), persisting borrelia potentially tolerant to antimicrobials have been detected in humans on multiple occasions by culture, microscopy, PCR, immunoassay, xenodiagnosis, and PCR/electrospray-mass spectrometry. The ability of a second course of antimicrobials to resolve clinical manifestations of Lyme arthritis in some patients also suggests the existence of persisting spirochetes in these patients. If PTLDS was found to be due to borrelial persistence in significant numbers of cases, therapies aimed at eliminating such organisms would be indicated and could be developed (1).
